# Long-term survival and transmission of *INI1*-mutation via nonpenetrant males in a family with rhabdoid tumour predisposition syndrome

**DOI:** 10.1038/sj.bjc.6604156

**Published:** 2007-12-18

**Authors:** A C J Ammerlaan, A Ararou, M P W A Houben, F Baas, C C Tijssen, J L J M Teepen, P Wesseling, T J M Hulsebos

**Affiliations:** 1 Department of Neurogenetics, Academic Medical Center, Amsterdam, The Netherlands; 2 Department of Neurology, St Elisabeth Hospital, Tilburg, The Netherlands; 3 Department of Pathology, St Elisabeth Hospital, Tilburg, The Netherlands; 4 Department of Pathology, Nijmegen Center for Molecular Life Sciences, Radboud University Nijmegen Medical Centre, Nijmegen, The Netherlands

**Keywords:** paediatric brain tumour, rhabdoid tumour predisposition syndrome, INI1, germline mutation, penetrance

## Abstract

Rhabdoid tumour predisposition syndrome (RTPS) is a rare syndrome caused by inheritance of a mutated *INI1* gene for which only two multigeneration families have been reported. To further characterise the genotype and phenotype of RTPS, we present a third family in which at least three cousins developed an atypical teratoid/rhabdoid tumour (AT/RT) at a young age. Two of these patients showed unusual long survival, and one of these developed an intracranial meningioma and a myoepithelioma of the lip in adulthood. Mutation analysis of *INI1* revealed a germline G>A mutation in the donor splice site of exon 4 (c.500+1G>A) in the patients and in their unaffected fathers. This mutation prevents normal splicing and concomitantly generates a stop codon, resulting in nonsense-mediated mRNA decay. Biallelic inactivation of *INI1* in the tumours, except for the meningioma, was confirmed by absence of nuclear INI1-protein staining. The myoepithelioma of one of the patients carried an identical somatic rearrangement in the *NF2* gene as the AT/RT, indicating that both tumours originated from a common precursor cell. In conclusion, this study demonstrates for the first time transmission of a germline *INI1-*mutation in a RTPS family via nonpenetrant males, long-term survival of two members of this family with an AT/RT, and involvement of *INI1* in the pathogenesis of myoepithelioma.

High-grade malignant brain tumours (MBTs) of childhood include medulloblastoma, primitive neuro-ectodermal tumour, anaplastic astrocytoma, glioblastoma, anaplastic ependymoma, atypical teratoid/rhabdoid tumour (AT/RT), and choroid plexus carcinoma (CPC) (Kalifa and Grill, 2005). Although most of these tumours are sporadic, familial cases have been described in which inherited germline mutations in tumour suppressor genes predispose to their development. Germline mutations in *PTCH* and *TP53* are known to be involved in the familial occurrence of medulloblastoma in childhood in Gorlin syndrome (Johnson *et al*, 1996) and of various types of brain tumours in Li-Fraumeni syndrome (Frebourg *et al*, 1995). The familial occurrence of ependymomas has been well documented, but the predisposing gene remains to be identified (Dimopoulos *et al*, 2006). Familial cases of AT/RT or CPC are very rare. Until now, two families have been described in which the development of these malignant childhood brain tumours correlated with the inheritance in two or more generations of a mutation in the *INI1/hSNF5/BAF47/SMARCB1* gene on chromosome 22 (Taylor *et al*, 2000; Janson *et al*, 2006). This tumour suppressor gene encodes INI1, which is a core subunit of the chromatin remodelling SWI/SNF complex (Wang *et al*, 1996; Phelan *et al*, 1999). In one family, an 18-month-old girl presented with a cerebellar rhabdoid tumour with an *INI1* exon 7 donor-splice site mutation, which she inherited from her unaffected carrier mother. A brother of the latter died at the age of 2 years of a tumour diagnosed as CPC with the same mutation (Taylor *et al*, 2000). In the other family, two half-brothers each developed an AT/RT at a young age and inherited an *INI1* insertion mutation in exon 4 from their unaffected carrier mother. Their maternal half-uncle, who was diagnosed with a medulloblastoma and a renal rhabdoid tumour, died at 2 years of age, suggesting that he inherited the same mutation, although this could not be studied (Janson *et al*, 2006).

Here, we present an extended molecular analysis of a previously reported family in which four cousins were affected with an MBT at young age (Hulsebos *et al*, 1999). These tumours were originally classified as anaplastic ependymomas (Nijssen *et al*, 1994). However, because of the previously documented involvement of chromosome 22 in the inheritance of the predisposition to these tumours (Hulsebos *et al*, 1999) and the possibility of misclassification, we investigated whether these anaplastic ependymomas were in fact AT/RTs. This proved to be the case, as we concluded from re-evaluation of their histopathology and from mutation and expression analysis of the *INI1* gene in three of these tumours. In addition, one of the patients developed a meningioma and a myoepithelioma in adulthood.

## PATIENTS AND METHODS

### Patient material

**Figure 1:**
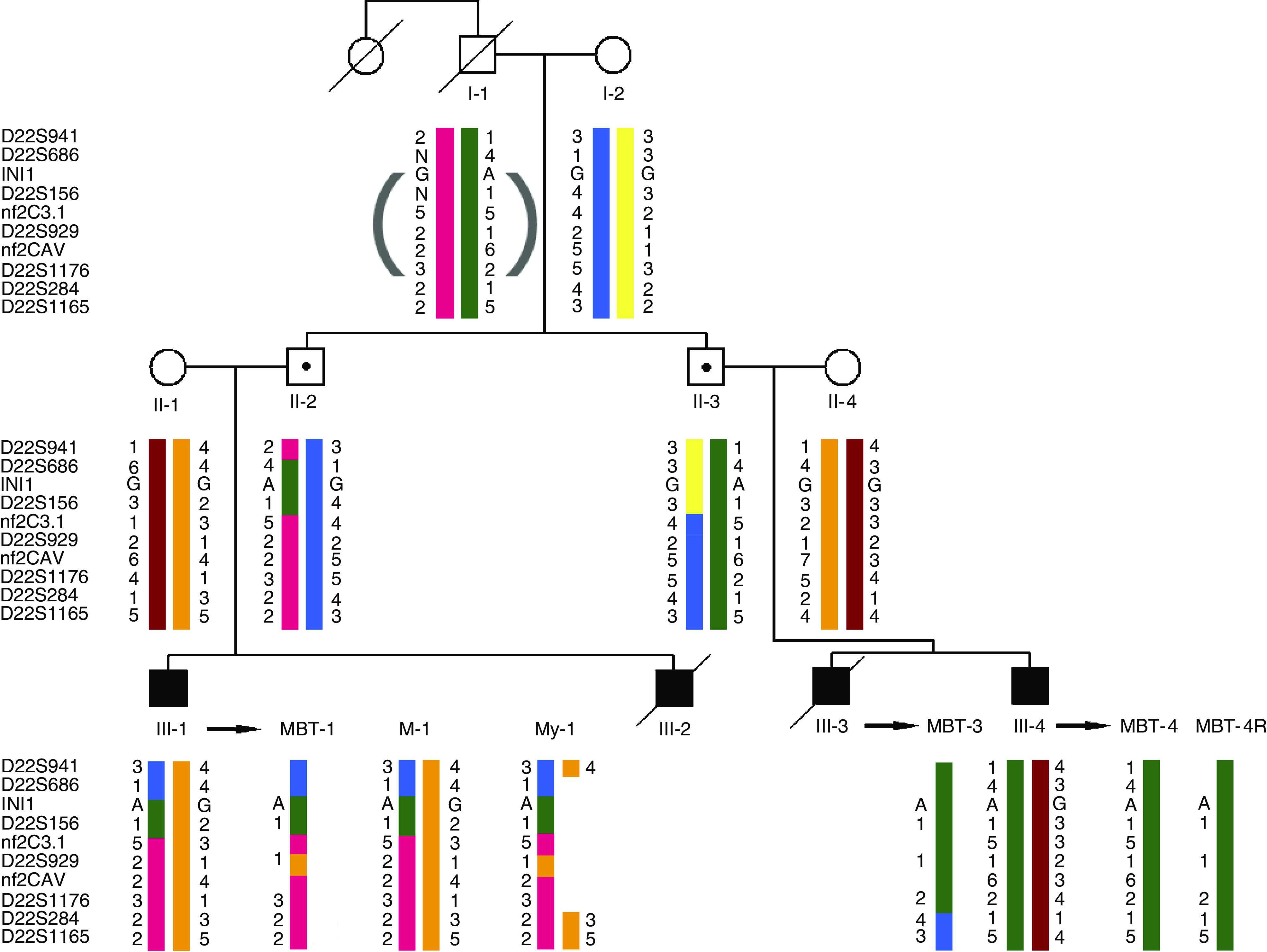
Haplotyping of family members and LOH analysis of tumours using microsatellite markers from chromosome 22. Patients with the germline *INI*-mutation are represented by black symbols and proven nonexpressing carriers of this mutation by dotted symbols. Haplotypes were constructed assuming minimal numbers of recombinations. Haplotypes for the grandfather (between brackets) were inferred. N: noninferred marker allele. MBT-1, MBT-3, and MBT-4(R) are the marker alleles retained in the MBT of patients III-1, III-3, and III-4 (R, recurrent tumour), respectively. M-1 and My-1 represent marker alleles retained in meningioma and myoepithelioma of patient III-1, respectively.

Written informed consent was obtained from patient III-1 (for pedigree see Figure 1). Each of the four cousins developed an MBT at a young age (<5 years). After removal of the tumour, patient III-1 received adjuvant chemotherapy (methotrexate, vincristine, and prednisolone) followed by craniospinal radiotherapy (3300 cGy in 22 doses) with a boost of 2100 cGy on the location of the tumour. He developed an intracranial meningioma and a myoepithelioma of the lip at, respectively, 25 and 26 years of age. Patient III-4 developed a recurrent brain tumour almost 2 years after removal of the primary brain tumour and treatment with chemotherapy (vincristine, procarbazine, and methotrexate). The sister of the paternal grandfather (I-1) of the four cousins died of an uncharacterised brain tumour at the age of 2 years. The clinical findings for the patients are summarised in Table 1.

Freshly frozen tumour tissue was only available from the meningioma of patient III-1 Formalin-fixed and paraffin-embedded tumour tissue was available from the other tumours.

### DNA and RNA samples

Tumour DNA was extracted using commercially available kits (QIAGEN, Venlo, The Netherlands). Extraction of constitutional DNA from blood leukocytes was performed as described previously (Hulsebos *et al*, 1999).

### Microsatellite analysis

Microsatellite markers used for haplotype analysis of family members and loss of heterozygosity (LOH) analysis in tumours were, ordered from 22cen to 22qter, D22S420, D22S427, D22S941, D22S311, D22S446, D22S539, D22S686, D22S303, D22S257, D22S345, D22S156, RH13801, D22S925, D22S926, D22S419, D22S421, D22S1164, D22S429, D22S1148, D22S310, D22S1167, D22S1163, D22S1150, nf2C3.1, D22S929, nf2CAV, D22S1176, D22S280, D22S284, D22S419, D22S1165, D22S1171, D22S274, D22S1169, D22S1145. Because of substantial degradation of template DNA, reliable determination of the allele status of markers generating polymerase chain reaction (PCR) products longer than 150 bp could not be performed for the MBTs of III-1 and III-3 and for the recurrent tumour of III-4. Primer sequences and amplification conditions were taken from the Genome Database at http://www.gdb.org/ or from Legoix *et al* (1999). Polymerase chain reaction reactions were performed essentially as described before (Bijlsma *et al*, 1995). ^32^P-dCTP-labelled PCR products were separated on 6% acrylamide denaturing gels and visualised with autoradiography.

### Mutation analysis

The *INI1* and *NF2* genes were sequenced by using genomic DNA as substrate for amplification by PCR. Primer sequences for mutation analysis of the 9 *INI1* exons and the 17 *NF2* exons have been given previously (Hulsebos *et al*, 2007). In degraded DNA samples, the exon 4–intron 4 junction region of *INI1* was sequenced using forward primer 5′-catgctccacaaccatcaac-3′ and reverse primer 5′-aactgaaacgtgctggagaac-3′, generating a PCR-product of 131 bp.

### Denaturing high-performance liquid chromatography

The exon 4–intron 4 junction region of *INI1* was amplified with forward primer 5′-ggatcaggtcctatactgac-3′ and reverse primer 5′-aactgaaacgtgctggagaac-3′, generating a product of 248 bp. Polymerase chain reaction products were analysed on an Agilent 1100 system (Agilent, Amstelveen, The Netherlands) equipped with a Helix DNA column (Varian, Middelburg, The Netherlands). Denaturing high-performance liquid chromatography (DHPLC) running conditions are available upon request.

### Multiple ligation-dependent probe amplification

Multiple ligation-dependent probe amplification (MLPA) analysis of the *NF2* gene was performed using the SALSA P044 NF2 MLPA Kit according to the instructions of the manufacturer (MRC-Holland, Amsterdam, The Netherlands).

### Immunohistochemical analysis

Immunohistochemical analysis of the INI1 protein was performed using a BAF47/INI1 antibody (BD Transduction Laboratories, Franklin Lakes, NJ, USA) on formalin-fixed, paraffin-embedded tumour tissue as described previously (Hulsebos *et al*, 2007).

## RESULTS

### Mutation analysis of INI1

**Figure 2:**
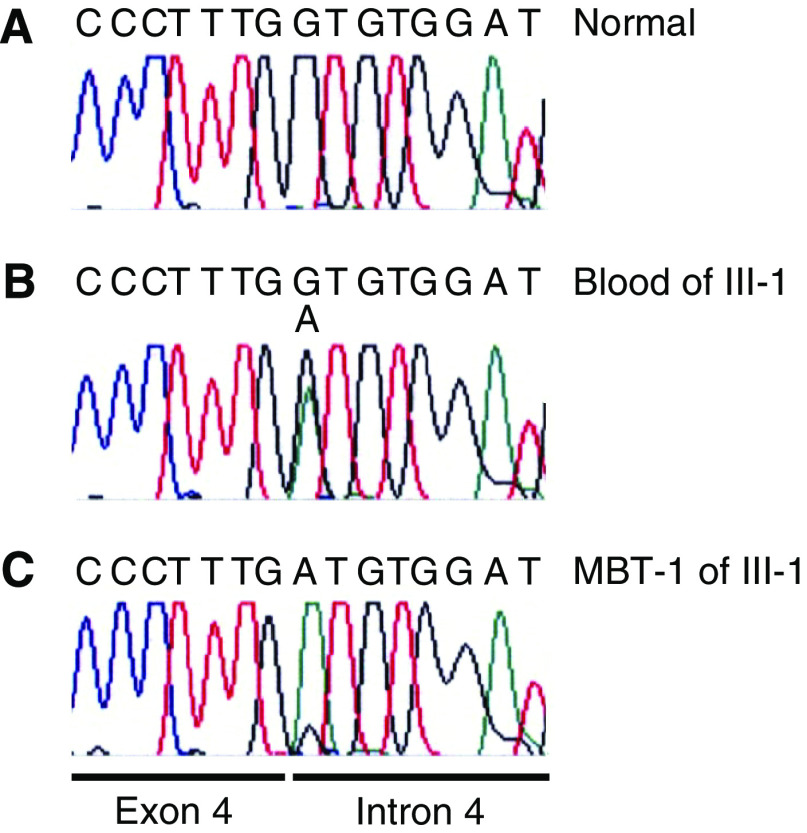
Sequence analysis of the exon 4–intron 4 boundary of *INI1*. Constitutional heterozygosity for the G>A mutation in blood DNA of patient III-1 (**B**) but not in normal DNA (**A**) and retention of the mutant A-allele in MBT DNA of patient III-1 (MBT-1) (**C**).

Sequencing of the 9 *INI1*-exons and their flanking sequences in the constitutional blood DNA of patient III-1 revealed a mutation in the donor splice site of exon 4 (Figure 2). This heterozygous G>A mutation alters the conserved GT sequence at the beginning of intron 4 (c.500+1G>A of the mRNA (GenBank accession number U04847)), enabling read-through of the transcript at this position. Concomitantly, the G>A mutation results in the generation of an in-frame stopcodon (UGG → UGA, p.W167X) in the read-through transcript. This mutation was also present in the constitutional DNA of patient III-4 and of the unaffected fathers II-2 and II-3. As exemplified in Figure 2 for tumour MBT-1, the malignant brain tumours of patients III-1(MBT-1), III-3 (MBT-3), and III-4 (MBT-4, MBT-4R) and the myoepithelioma of patient III-1 (My-1), but not his meningioma (M-1), showed loss of the wild-type G-allele and retention of the mutant A-allele. Mothers II-1 and II-4 and also grandmother I-2 had only the wild-type sequence in their constitutional DNA (data not shown). The sequencing data for *INI1* are summarised in Figure 1. To exclude that the G>A donor splice site mutation represents a polymorphism, we screened the constitutional DNA of 132 normal individuals (264 chromosomes 22) by DHPLC analysis of PCR products containing the exon 4–intron 4 junction region in *INI1* but found no mutation-specific profile.

### Immunohistochemical analysis of INI1 protein expression

**Figure 3:**
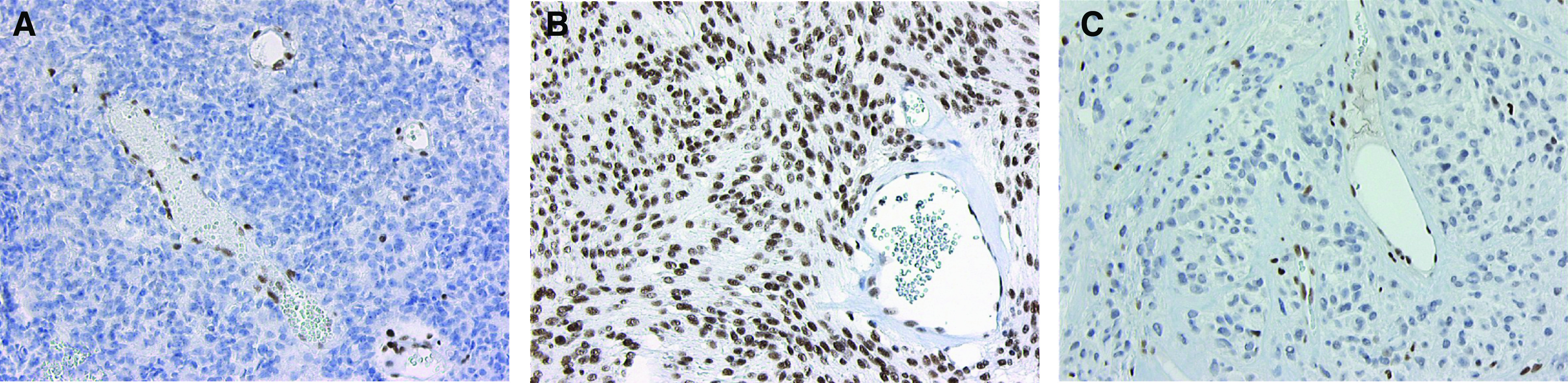
Immunohistochemical INI1-staining of the recurrent MBT of patient III-4 (MBT-4R) (**A**) and the meningioma (M-1) (**B**) and myoepithelioma (My-1) (**C**) of patient III-1. Note absence of nuclear staining of tumour cells in MBT-4R and myoepithelioma. In contrast, the nuclei of endothelial cells of the blood vessels in both tumours and the nuclei of all cells in the meningioma show unequivocal staining. Original magnification × 200.

In about 3% of cases, the normal GT donor splice site signal at the exon 4–intron 4 junction is ignored during transcription of *INI1* and splicing occurs at a cryptic splice site (GC), which is 54 bp distal to the normal splice site (GenBank accession number AK024025) (Favre *et al*, 2003). Mutation of the GT donor splice site may increase the rate of this alternative splicing. However, the concomitant generation of a stop-codon confers the alternatively spliced transcript in an excellent target for nonsense-mediated decay (Nagy and Maquat, 1998), resulting in absence of INI1 protein encoded by the mutated allele. Alternatively, the mutation may result in skipping of exon 4, generating a transcript that is 138 bases shorter than the wild-type transcript and an INI protein with an in-frame deletion of 46 amino-acid residues. To determine the presence of INI1 protein in the tumours, we performed immunohistochemistry with an INI1-antibody on available histological sections of tumours. The antibody is directed against the C-terminal part of the protein (amino-acid residues 257–359), which remains intact after the eventual in-frame deletion of 46 amino-acid residues (nrs122–167) resulting from exon-4 skipping. As shown in Figure 3, nuclear staining was absent in tumour cells of the recurrence of the brain tumour of patient III-4 (MBT-4R) and in the myoepithelioma of patient III-1 (My-1), but abundantly present in all cells of the meningioma (M-1) of patient III-1. As our sequencing data demonstrated that the normal *INI1* gene was lost in the recurrent brain tumour and in the myoepithelioma, but remained in the meningioma, this staining pattern is consistent with the notion that the mutant allele does not encode INI1 protein or shortened INI1 protein as the consequence of exon-4-skipping.

### Microsatellite analysis with chromosome 22 markers

**Figure 4:**
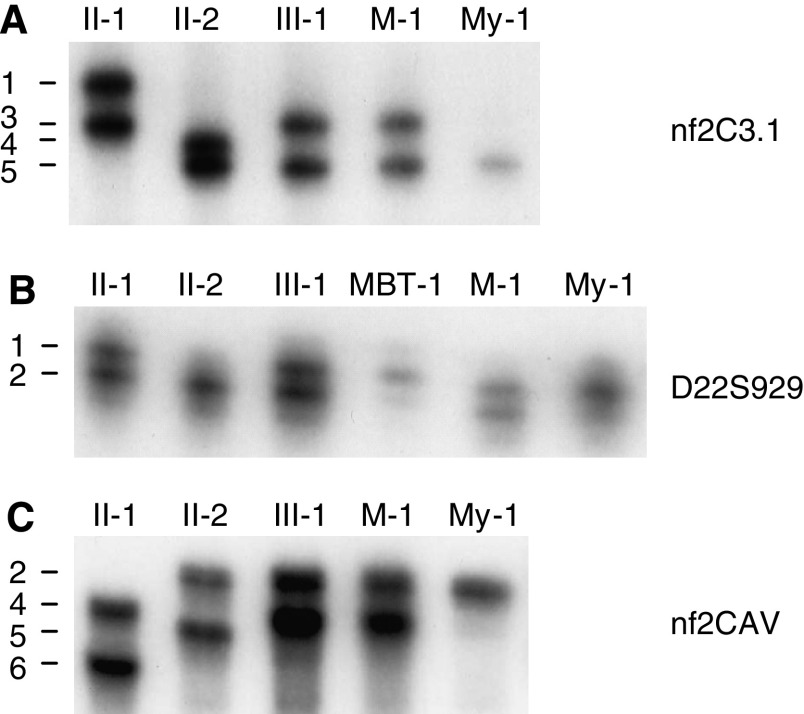
Microsatellite analysis with intragenic *NF2* markers nf2C3.1 (**A**) *D22S929* (**B**) and nf2CAV (**C**) of constitutional DNAs of patient III-1, mother II-1, and father II-2, and of tumour DNAs of patient III-1. Positions of alleles are indicated on the left. MBT-1, M-1, and My-1 represent retained alleles in the MBT, meningioma, and myoepithelioma of patient III-1, respectively.

Our sequencing data indicated that the normal INI1 gene was lost in all analysed tumours, except for the meningioma. To determine the mechanism by which these losses occurred, we extended previously reported haplotype and LOH analyses (Hulsebos *et al*, 1999). The key genetic events are summarised in Figure 1. The LOH analyses indicate that loss of the normal INI1 gene is caused by the deletion of a complete copy of chromosome 22 in the MBTs, but by interstitial deletion in the myoepithelioma. Furthermore, we noted the aberrant retention of allele 1 of marker D22S929 in the brain tumour as well as in the myoepithelioma of patient III-1 (see Figure 1, Figure 4). Flanking markers nf2C3.1 and nf2CAV, however, showed normal retention of the paternal alleles in the myoepithelioma of this patient. As the three markers are located within the NF2 gene (Legoix *et al*, 1999), we investigated whether this gene was affected by the rearrangement. Sequencing of the 17 exons of NF2 in constitutional, meningioma, and myoepithelioma DNA of patient III-1 revealed no mutations. Multiple ligation-dependent probe amplification analysis of the NF2 gene demonstrated no additional copy number aberrations in blood or tumours of this patient (data not shown).

## DISCUSSION

### Inheritance of the INI1-mutation

We have shown by sequence analysis that the unaffected fathers of the patients were carriers of the germline mutation. As the paternal grandfather's sister died of a brain tumour at 2 years of age and his sons transmitted the mutation, it is most likely that the grandfather is also an unaffected carrier and that his sister developed the brain tumour because of inheritance of the *INI1*-mutation. This is also supported by the deduced haplotypes of the grandfather's copies of chromosome 22. This study thus documents the inheritance of a germline *INI1*-mutation that predisposes to the development of MBTs at young age in three subsequent generations of a family. Inheritance of an *INI1*-mutation via asymptomatic carriers has been reported before in two other families with childhood brain tumours (Taylor *et al*, 2000; Janson *et al*, 2006). Although the analyses of the latter families suggested that the asymptomatic transmission of the *INI1*-mutation exclusively occurred via female carriers, it is clear from our family that the transmission can as well take place via healthy male carriers.

### Malignant brain tumours are AT/RTs

Originally, based on their (immuno)histological, ultrastructural, and clinical characteristics, the MBTs of the patients in our family were classified as anaplastic ependymomas (Nijssen *et al*, 1994). Here, we demonstrate the biallelic inactivation by mutation and deletion of *INI1* in the MBTs of patients III-1, III-3, and III-4 and the absence of INI1-protein expression in the recurrence of patient III-4 (Figure 2, Figure 3, respectively). *INI1* mutations have not been described in ependymomas (Sevenet *et al*, 1999a; Kraus *et al*, 2001; Weber *et al*, 2001), whereas biallelic inactivation of *INI1* and loss of INI1 protein expression are characteristic features of AT/RTs (Versteege *et al*, 1998; Judkins *et al*, 2004; Biegel, 2006). Moreover, re-evaluation revealed rhabdoid cells in histological sections of the brain tumours (Table 1). Our data thus show that the brain tumours in at least three of the four cousins are in fact AT/RTs. Especially, as correct histopathological classification of paediatric brain tumours can be challenging (Judkins *et al*, 2005; Haberler *et al*, 2006), our present findings suggest that testing for the mutational status of *INI1* is warranted to exclude rhabdoid tumour predisposition syndrome (RTPS) in previous (Dimopoulos *et al*, 2006) and future reports on familial ependymoma.

### INI1 inactivation in the tumours of patient III-1

Patient III-1 developed an intracranial meningioma and a myoepithelioma of the upper lip, respectively, 21 and 22 years after operation and chemo- and radiotherapy for the AT/RT.

The meningioma had no mutation in the *NF2* gene and displayed a complex karyotype, as evidenced by single-nucleotide polymorphism analysis, including extensive losses on chromosome arm 1p, but no loss of chromosome 22 (data not shown). These molecular and also the clinical characteristics of the meningioma strongly suggest that the development of this tumour is radiation-induced and not part of the RTPS. The biallelic inactivation of *INI1* and loss of INI1 protein-expression in the myoepithelioma is remarkable. This benign tumour type is generally characterised by a paucity of genetic alterations and involvement of chromosome 22 or *INI1* in the development of this tumour has never been reported before (Hungermann *et al*, 2002).

### NF2 rearrangement in the tumours of patient III-1

Another remarkable finding is the aberrant retention of the maternal allele 1 of marker *D22S929* in *NF2* on the paternal copy of chromosome 22 that remained in the AT/RT as well as in the myoepithelioma of patient III-1 (Figure 1, Figure 4). This might be explained by assuming a somatic double recombination (or gene conversion) event in a common precursor cell for both tumours, substituting allele 2 for allele 1 on the paternally derived chromosome 22. Although we were unable to demonstrate that the recombination event directly affected the coding regions of *NF2*, we nevertheless conclude that both tumours must have a common origin. Several patients with a constitutional *INI1* mutation and with two tumours at different sites have been reported (Sevenet *et al*, 1999b; Savla *et al*, 2000; Kusafuka *et al*, 2004; Biegel, 2006; Giunti *et al*, 2006; Meyers *et al*, 2006). It will be of interest to determine whether the tumours of these patients, like those of patient III-1, are primary tumours, share somatic changes in the genome, and originate from a common precursor cell as well.

In conclusion, we have documented the variable expression and incomplete penetrance of an *INI1* germline mutation in a third multigeneration family with the RTPS (Wesseling *et al*, 2007). Inheritance of the mutation resulted in the development early in life of an AT/RT in at least three cousins. Two of these patients survived this tumour for over 15 years and one of these developed a (probably radiation induced) meningioma and a myoepithelioma in adulthood. We recently showed (Hulsebos *et al*, 2007) that *INI1* is a predisposing gene in familial schwannomatosis, a disorder in which patients develop multiple schwannomas later in life and that is also characterised by variable expression and incomplete penetrance (MacCollin *et al*, 2003). It has been hypothesised that in patients with a germline *INI1* mutation, a developmental window exists at young age in which most rhabdoid tumours occur (Janson *et al*, 2006). Carriers of the *INI1* mutation that do not develop such a tumour at young age might be at increased risk of developing other *INI1*-related, but not necessarily malignant tumours later in life.

Given the small number of splice site mutations in malignant rhabdoid tumours reported to date (Biegel, 2006), it is interesting to note that our family and one other multigeneration family with RTPS (Taylor *et al*, 2000) demonstrate an *INI1* mutation at a splice site, raising the possibility that carriers of such a mutation may be less likely to be affected. In case of our family, there is the additional possibility that in the brain of the unaffected carriers alternative splicing of the INI1-mutant allele transcript, leading to absence of INI1 protein in the brain tumours of the patients, does not occur. Instead, skipping of exon 4, resulting in the in-frame deletion of 46 amino-acid residues in the INI1 protein, may occur. This truncated protein may still be (partly) functional, at least not contributing to the development of brain tumours in these carriers. Finally, whereas long-term survival in children with AT/RT is extremely rare (Tekautz *et al*, 2005; Squire *et al*, 2007), two of the four cousins we described survive for more than 15 years now. More multigeneration families with the RTPS need to be investigated to further elucidate genotype–phenotype correlations in this syndrome.

## Figures and Tables

**Table 1:** Clinical and histopathological features of patients with malignant brain tumour of infancy

**Patient**	**Current diagnosis (ID in Figure 1)**	**Age (years) at diagnosis**	**Survival (years)**	**Location**	**Rhabdoid cells^a^**	**INI1 expression^b^**
III-1	AT/RT (MBT-1)	4.5	Alive and well at age 29	Fourth ventricle	++	ND
	Meningothelial meningioma (M-1)	25		Left temporal	−	+
	Myoepithelioma (My-1)	26		Upper lip	−	−
III-2	AT/RT?	0.6	Death of disease at age 0.6	Fourth ventricle	ND	ND
III-3	AT/RT (MBT-3)	1.7	Death of disease at age 2.0	Right lateral ventricle	++	ND
III-4	AT/RT (MBT-4)	0.6	Alive and well at age 16	Fourth ventricle	+	ND
	AT/RT recurrence (MBT-4R)	2.5		Fourth ventricle	+	−

a Presence (+) or absence (−) of cells with eosinophilic intracytoplasmic globules; these cells were generally vimentin and GFAP positive; additionally, the cells showed variable EMA staining, but were desmin negative.

b +=retention; −=loss; ND=not determined (no tissue available anymore).
